# Ets1 Induces Dysplastic Changes When Expressed in Terminally-Differentiating Squamous Epidermal Cells

**DOI:** 10.1371/journal.pone.0004179

**Published:** 2009-01-14

**Authors:** Priyadharsini Nagarajan, Neha Parikh, Lee Ann Garrett-Sinha, Satrajit Sinha

**Affiliations:** Department of Biochemistry, State University of New York at Buffalo, Center for Excellence in Bioinformatics and Life Sciences, Buffalo, New York, United States of America; University of Helsinki, Finland

## Abstract

**Background:**

Ets1 is an oncogene that functions as a transcription factor and regulates the activity of many genes potentially important for tumor initiation and progression. Interestingly, the Ets1 oncogene is over-expressed in many human squamous cell cancers and over-expression is highly correlated with invasion and metastasis. Thus, Ets1 is believed to mainly play a role in later stages of the oncogenic process, but not early events.

**Methodology/Principal Findings:**

To better define the role of Ets1 in squamous cell carcinogenesis, we generated a transgenic mouse model in which expression of the Ets1 oncogene could be temporally and spatially regulated. Upon Ets1 induction in differentiating cells of stratified squamous epithelium, these mice exhibited dramatic changes in epithelial organization including increased proliferation and blocked terminal differentiation. The phenotype was completely reversed when Ets1 expression was suppressed. In mice where Ets1 expression was re-induced at a later age, the phenotype was more localized and the lesions that developed were more invasive. Many potential Ets1 targets were upregulated in the skin of these mice with the most dramatic being the metalloprotease MMP13, which we demonstrate to be a direct transcriptional target of Ets1.

**Conclusions/Significance:**

Collectively, our data reveal that upregulation of Ets1 can be an early event that promotes pre-neoplastic changes in epidermal tissues via its regulation of key genes driving growth and invasion. Thus, the Ets1 oncogene may be important for oncogenic processes in both early and late stages of tumor development.

## Introduction

The stratified squamous epithelium of the skin forms a barrier between the underlying tissues and the outer milieu to prevent the passage of water and other substances between these compartments. Keratinocytes are the principal cell type found in stratified squamous epithelia and generate biomolecules that are necessary for the stability and resistance of the epithelial layer to mechanical stress. The innermost layer of this stratified epithelium, known as the basal layer (stratum basale), consists of a proliferative compartment of undifferentiated cells. Basal cells periodically withdraw from the cell cycle, detach from the basement membrane and migrate outwards to enter the suprabasal compartment. Differentiation of keratinocytes can be monitored by the morphological appearance of the cells and by the expression of particular marker proteins. Based on these criteria, the differentiated layers of the epidermis can be visualized as three separate regions: the spinous or prickle cell layer (stratum spinosum), the granular layer (stratum granulosum) and the cornified layer (stratum corneum).

Squamous cell carcinoma (SCC) is a malignant tumor of skin keratinocytes that frequently arises in response to excessive sun exposure or to chronic irritation. Numerous mouse models of squamous cell cancer have been developed including carcinogen-induced SCC, ultraviolet (UV) light-induced SCC and spontaneous SCC in various transgenic and knockout mouse strains. Several common genetic alterations have been detected in squamous cell tumors, including upregulation of oncogenes and mutation of tumor suppressor genes [1], [2]. In addition, several recent studies have reported upregulation of the Ets1 proto-oncogene in human SCC arising in skin [3] and other stratified epithelia [4], [5], [6], [7], [8]. Moreover, upregulation of Ets1 expression has also been detected in animal models of oral SCC [9], [10]. Expression of high levels of Ets1 is correlated with increased invasiveness and metastatic potential in both human SCC and animal models of SCC.

Ets1 is a transcription factor that regulates the expression of many key genes that are involved in cell growth, survival and invasion. Importantly, Ets1 is thought to regulate the expression of proteases such as urokinase plasminogen activator (*uPA*) and various members of the matrix metalloprotease family (*MMP1*, *MMP2*, *MMP3*, *MMP9* and *MMP13*) [11], [12], [13], [14], [15], [16]. Expression of these proteases is likely required for tumor cells to degrade the surrounding extracellular matrix, invade nearby tissues and eventually metastasize to distant sites. In addition, to these proteases, Ets1 is also implicated in upregulation of a wide-variety of genes that may promote tumorigenesis including genes that control cellular proliferation or survival (*bax*, *bcl-2*, *caspase-1*, *c-myc*, *CDK11*, *Fas ligand*, *GADD153*, *JunB*, *mdm2*, *p16*, *p21* and *p53*) [17], [18], [19], [20], [21], [22], [23], [24], [25], [26], [27], [28] and genes involved in responses to a hypoxic environment [29]. Furthermore, Ets1 has been reported to directly interact with the tumor suppressor protein p53 and modulate its transcriptional activity [30], [31], [32], [33], [34].

Because Ets1 regulates the expression of a large cohort of target genes that influence cellular proliferation and survival, Ets1 might play an important role in early stages of the carcinogenic process in addition to its better known role in tumor invasion and metastasis. To investigate the ability of Ets1 to drive neoplastic or pre-neoplastic changes in stratified squamous epithelial cells, we developed a transgenic mouse model that allows us to inducibly express high levels of Ets1 protein in skin epithelium. Using this inducible model system, we have explored the role of Ets1 in SCC.

## Results

### Ets1 is expressed predominantly in the proliferative basal layer of the stratified squamous epithelium

**Figure 1:**
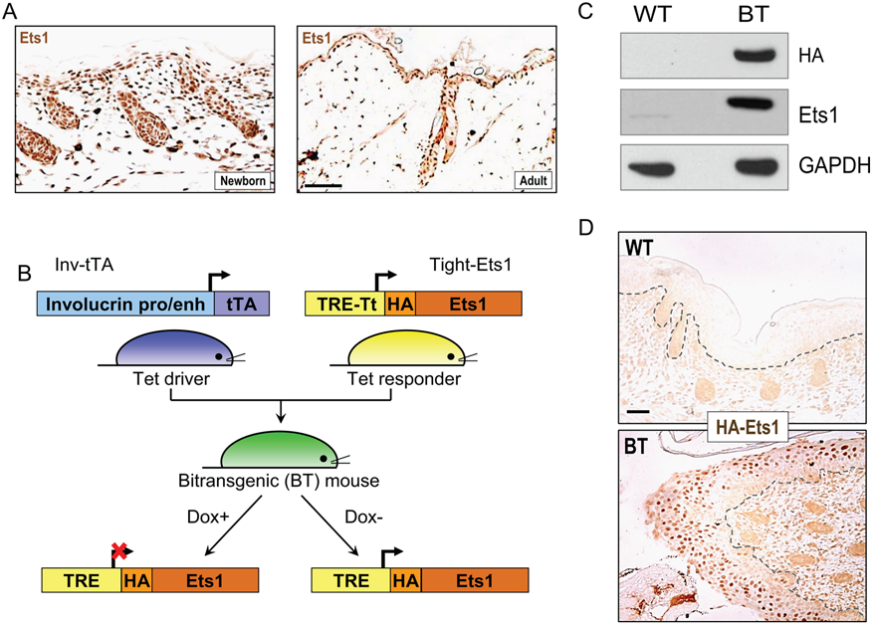
Development of an inducible transgenic model to express Ets1 in differentiated epidermal keratinocytes. (A) Immuno-histochemical localization of Ets1 in the nuclei of epidermal keratinocytes in newborn and adult mice. (B) Schematic representation of the tetracycline-inducible Ets1 transgenic system. The system consists of two separate transgenes- the Tet driver transgene in which the tetracycline-regulated transactivator protein (tTA) is under the control of the involucrin promoter and enhancer elements and the Tet responder transgene in which an HA-tagged version of murine Ets1 is under the control of the pTight promoter containing tet-responsive elements (TRE, which are tTA binding sites). When Tet driver and Tet responder mice are crossed, BT offspring are produced. Maintaining these BT animals on Dox supplementation inhibits transgene expression, whereas withdrawal of Dox supplementation causes induced expression of the Ets1 transgene in cells that normally express involucrin (upper spinous and granular layers). (C) Western blot analysis of transgene expression in skin extracts from newborn wild-type and BT mice using anti-Ets1 and anti-HA antibodies. GAPDH serves as a loading control. (D) Immuno-histochemical localization of transgene expression using antibody against the HA epitope tag.

Although Ets1 has previously been reported to be expressed in embryonic mouse skin [35], the exact expression pattern of this transcription factor during epidermal keratinocyte differentiation has not been determined. We performed immunostaining for Ets1 protein in the skin epithelium of newborn and adult mice, which showed that expression of Ets1 is predominantly restricted to the nuclei of proliferative basal layer keratinocytes of the epidermis and hair follicles of newborn mice (Fig. 1A). In contrast, more differentiated suprabasal cells expressed little or no Ets1 protein. A similar pattern of expression was detected in adult mice, with the majority of Ets1 positive cells being localized to the basal layer and to the hair follicles (Fig. 1A). We conclude that in mice Ets1 is mainly expressed in undifferentiated keratinocytes of the skin and its expression is downregulated as cells commit to the terminal differentiation program. A similar pattern of expression of Ets1 in basal cells, but not suprabasal differentiated cells, has been detected in human skin samples [3].

### Development of an inducible Ets1 transgenic model

As discussed in the Introduction, high levels of Ets1 expression have been detected in SCC in humans and rodents. These observations along with the expression pattern of Ets1 in the proliferative compartment of the skin suggest that Ets1 may play a role in keratinocyte proliferation. However, Ets1 knockout mice do not display any obvious defects in the proliferation or differentiation of skin keratinocytes, although they are characterized by a previously-unreported white-spotted coat color, suggesting a defect in melanocyte proliferation, survival or migration (Fig S1A–C). The lack of an obvious keratinocyte phenotype in Ets1 knockout mice may be due to functional compensation by other Ets family members, a number of which are expressed in skin. Indeed, the closely related Ets2 protein is highly expressed in skin and has been shown to play a role in hair follicle morphogenesis [36].

Although Ets1 is not essential for epidermal homeostasis under physiological conditions, it may contribute to oncogenic transformation of these cells by promoting proliferation, epithelio-mesenchymal transition (EMT) and/or invasion and metastasis. In order to study the function of the Ets1 oncogene in skin carcinogenesis, we developed a Tet-inducible Ets1 transgenic mouse model. The full-length murine Ets1 cDNA was cloned in-frame with an N-terminal HA epitope tag and inserted downstream of the doxycycline-regulated pTight promoter in the vector pTRE-Tight to generate the construct pTight-Ets1 (Figure 1B). The pTight-Ets1 construct was micro-injected into fertilized mouse oocytes and six transgenic founders were obtained. To test the ability of the six transgenic lines to inducibly express Ets1, we crossed each founder with previously-described transgenic mice expressing the tetracycline-transactivator (tTA) under the control of the human involucrin promoter and enhancer elements (INV-tTA mice) [37]. In the resulting bitransgenic (BT) offspring (pTight-Ets1/ INV-tTA), HA-Ets1 can be induced in the upper spinous and granular layers of skin and other stratified squamous epithelial tissues when doxycyline (Dox) is withheld. Note that this induced Ets1 expression pattern is opposite to the normal pattern of Ets1 gene expression in the basal layer of the epidermis.

When Ets1 expression was induced during embryonic development by withholding Dox, BT newborn animals derived from two of the six pTight-Ets1 founder animals, line C and E exhibited a dramatic skin phenotype leading to a defect in epidermal barrier formation and neonatal lethality (data not shown). In agreement with the skin phenotype, line C and E were shown to express the HA-Ets1 transgene by Western blotting (Figure 1C and data not shown). The majority of the studies reported herein were performed using the transgenic line C, but similar results were obtained with line E.

To determine whether the transgene was faithfully expressed in the differentiated layers of the epidermis, immunohistochemical analysis using anti-HA antibodies was carried out. As shown in Figure 1D, HA staining was evident in the differentiated layers of the skin epithelium, but not in the undifferentiated basal layers. The Ets1 transgene would also be expressed in differentiated cells of the oral mucosa and other internal, stratified epithelia (i.e., esophagus, forestomach and anogenital linings) since the involucrin promoter has been shown to be active in these sites [37]. Transgene expression could be suppressed by supplementation with Dox as expected and mice maintained on Dox during embryonic development and in the post-natal period were normal at birth, did not have an epidermal barrier defect and survived to adulthood. Thus, we have developed a Doxycycline–regulated, inducible transgenic model system that allows us to turn Ets1 expression on or off in the stratified squamous epithelia of mice at different ages and for different periods of time.

### Expression of the Ets1 transgene in adult mice leads to conspicuous changes in the skin

**Figure 2:**
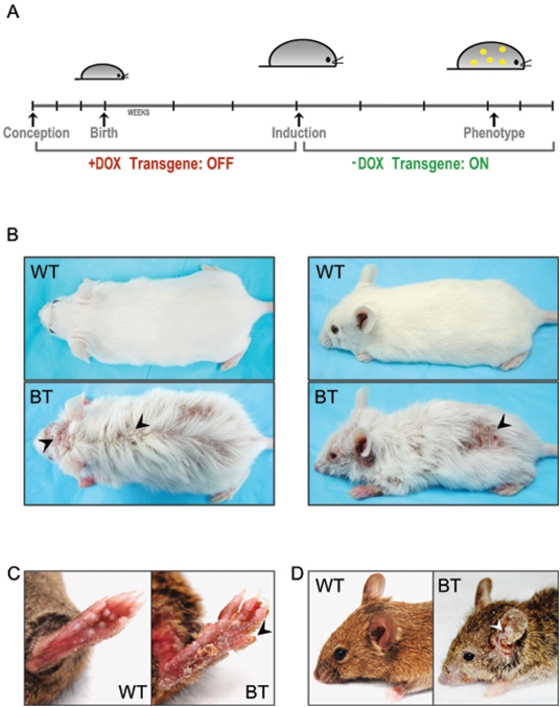
Ets1 expression results in dramatic alterations to skin homeostasis. (A) Time course of transgene induction. Ets1 expression is suppressed prenatally and postnatally by feeding the mothers a Dox-containing diet. After weaning at the age of 3 weeks, the Ets1 transgene is induced by switching the mice to a diet lacking Dox. The phenotype can be observed within 3–6 weeks after diet change. (B–D) Gross phenotype of several sets of adult WT and BT mice. Arrowheads point to sores, ulcers and crusting on the skin and paws of BT mice. Note that BT mice are substantially thinner than their wild type littermates.

As noted above, induction of Ets1 expression during embryonic development leads to neonatal lethality due to a failure to establish a normal skin barrier function. To overcome this problem, we suppressed expression of the Ets1 transgene during the embryonic and postnatal period by maintaining the mice on Dox supplementation. After weaning, Dox was withdrawn allowing transgene expression (Figure 2A). In adult BT mice, obvious epidermal alterations became evident within a three- to six-week period after Dox withdrawal (Figure 2B–D). In particular, BT mice exhibited patchy hair loss, crusting, scaly skin and open sores covering the body surface. The skin of the paws and tail was frequently affected. In addition, BT mice were often very thin and wasted compared to control littermates, potentially due to difficulties in feeding caused by changes to the oral cavity, esophagus or forestomach. We noted some variability in the severity of the phenotype of induced mice, this might reflect differences in genetic background modifiers as the pTight-Ets1 transgenic mice were initially derived from a mixed C3H×C57BL/10 genetic background and subsequently crossed to FVB/N mice. Alternatively, the differences in phenotype might reflect variegation in transgene expression or in the precise timing of transgene induction. The studies described below assessing histological changes in the skin epidermis were performed using samples from mice that displayed a visible phenotype after 3–6 weeks of Ets1 induction.

**Figure 3:**
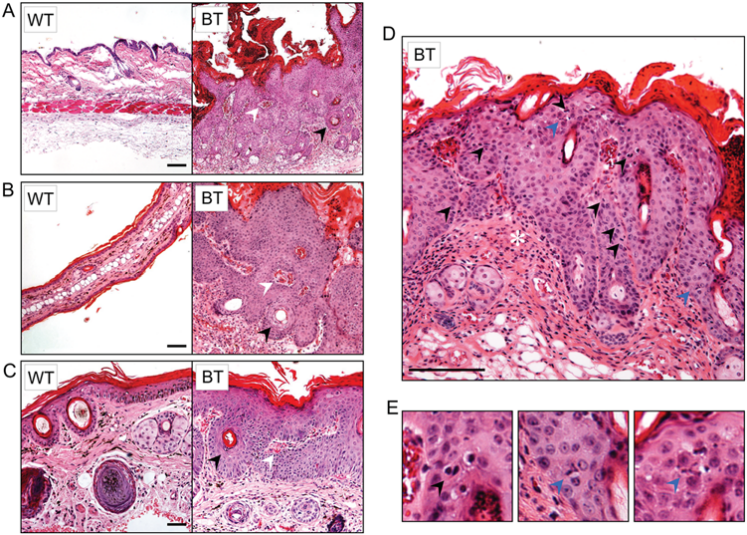
Ets1 expression causes hyperplastic and dysplastic changes in the skin epidermis. Microscopic features of the cutaneous changes in (A) dorsal trunk, (B) ear and (C) tail skin of BT mice upon Ets1 induction show epidermal and sebaceous hyperplasia, hyperkeratosis, and parakeratosis, keratin pearls (black arrowheads), vascularization, leukocytic infiltration and dermal enclosures within the epidermal downgrowths (white arrowheads). (D) High power view of the dorsal epidermis demonstrating the presence of several mitotic figures including some in the suprabasal layers (black arrowheads). Reactive leukocytic infiltration is also evident (asterisk). (E) Further magnification of supra-basal mitoses in lesions of the dorsal skin in BT mice. Some of the mitoses exhibit abnormal, tripolar mitotic spindles (blue arrowheads).

Histological examination of BT mouse skin revealed striking hyperplastic and dysplastic alterations when compared to single transgenic or non-transgenic control mice. The epidermal changes were most prominent in sections of the affected dorsal trunk, ear and tail skin (Figure 3A–C). The BT epidermis was dramatically thickened and hyperkeratotic when compared to control mice and was composed of predominantly large, pleomorphic cells with eosinophilic cytoplasm, suggesting that the expanded cell population was spinous in nature (acanthosis). Some keratinocytes of the stratum corneum, though cornified, retained nuclei (parakeratosis) indicating a partial block to terminal differentiation. The alteration of the normal pattern of keratinocyte differentiation was also apparent from the presence of keratin pearls scattered throughout the skin. In addition, there was an extensive invasion of the underlying dermis by the hyperplastic epidermal cells. The keratinocytes invaded the dermis in the form of large cohesive masses or thick downgrowths (10–20 cells across) often enclosing islands of dermal tissues within them. In some cases, enlarged sebaceous glands and the proximal end of hair follicles formed the leading edge of the invasive front. As a secondary response to the epidermal alterations, inflammatory reaction typified by leukocytic infiltration was also observed. Closer examination showed an increase in nuclear-to-cytoplasmic ratio, hyperchromatic nuclei and prominent nucleoli in the keratinocytes (Figure 3D). The proliferative capacity of the affected epidermis was augmented as evidenced by increased number of mitotic cells. Interestingly, these actively dividing cells were not restricted to the proliferative basal compartment of the epidermis, but were present in the suprabasal layers too. This suggests that some partially-differentiated keratinocytes retained or acquired proliferative capability in the supra-basal layers. A number of these dividing cells had atypical (tripolar) mitotic spindles, a feature often associated with transformation (Fig. 3E). Therefore, induction of Ets1 in differentiating keratinocytes leads to extensive hyperplasia associated with dysplastic differentiation and downgrowths of chords of keratinocytes into the dermal compartment.

### The keratinocyte differentiation program is disrupted in epithelia expressing Ets1

**Figure 4:**
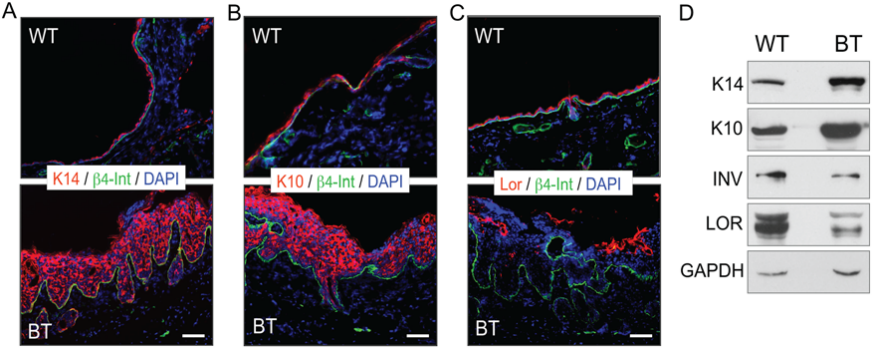
Ets1 expression disrupts the normal differentiation pattern of the skin epidermis. Immunofluorescent staining of epidermal differentiation markers on dorsal skin of induced Ets1 BT mice and littermate wild type mice. Each section was stained with antibodies to β4- integrin to mark the position of the basement membrane and with DAPI to detect nuclei. Epidermal differentiation was assessed by staining for (A) the basal layer marker keratin 14 (K14), (B) the spinous layer marker keratin 10 (K10) and (C) the granular layer marker loricrin (Lor). (D) Western blot analysis of dorsal skin extracts to confirm results obtained in immunofluorescence. GAPDH serves as a loading control for Western blots.

The histological analyses described above suggested that the normal differentiation program of epidermal keratinocytes was disrupted by expression of Ets1 in the differentiating layers of the skin. To confirm this result, we stained sections of the dorsal skin of BT and control mice with antibodies specific for marker proteins associated with particular differentiated layers of the epidermis. As shown in Figure 4, keratinocytes in the skin of BT mice demonstrated significant alterations in the expression of these markers. In particular, expression of cytokeratins typically localized to the basal layer (K5 and K14) was expanded such that most of the cells in the epidermis stained positive for these proteins (Figure 4A and data not shown). In addition, most suprabasal cells in the epidermis of BT mice expressed K10, a cytokeratin normally restricted to the spinous layer (Figure 4B). In contrast, the expression of markers such as loricrin, filaggrin and involucrin, which are normally restricted to the granular layer of the epidermis, was decreased (Figure 4C and data not shown). These results were confirmed by Western blotting (Figure 4D and data not shown). The reduced expression of loricrin, filaggrin and involucrin in skin of BT mice likely results in abnormal barrier function in the affected epidermis. In summary, these studies indicate that epidermal differentiation of keratinocytes in BT mice is disrupted with an expansion of keratinocyte populations expressing early differentiation markers and a reduction in keratinocyte populations expressing late differentiation markers.

### Keratinocytes in epithelia expressing Ets1 undergo enhanced proliferation and induce the growth of local blood vessels

**Figure 5:**
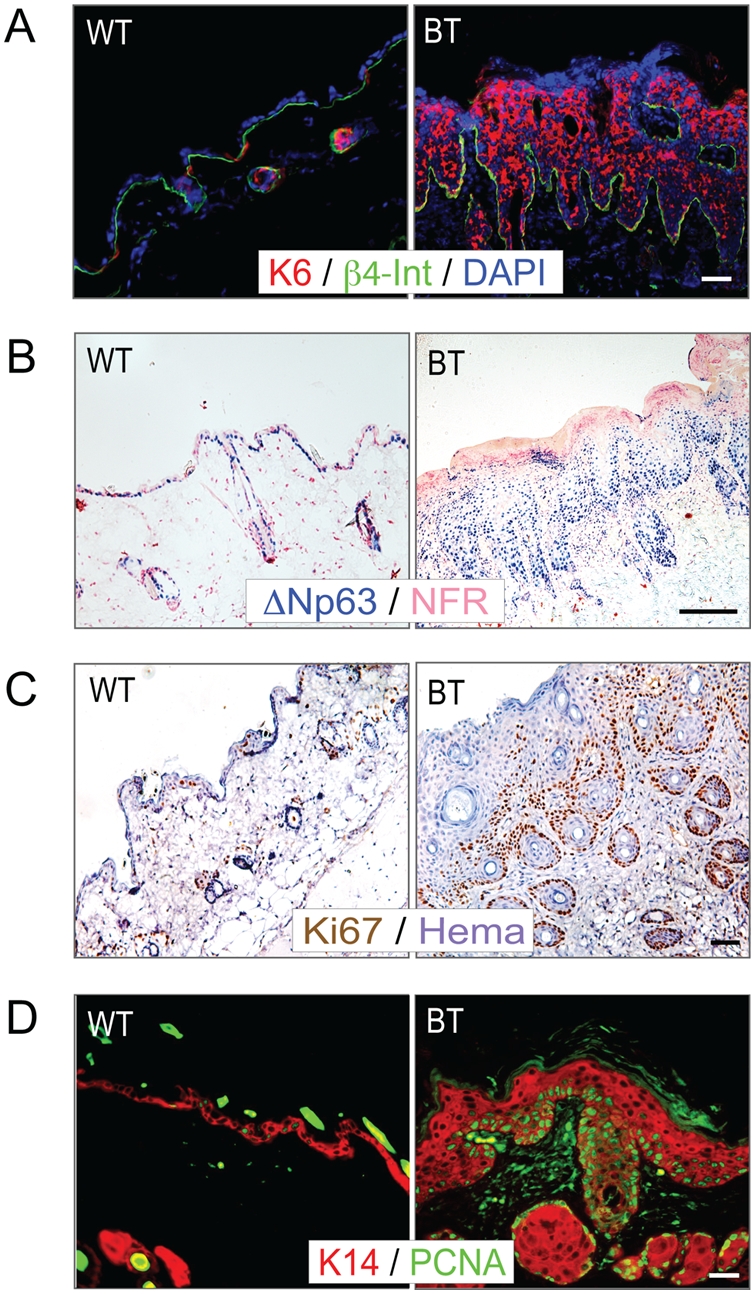
Ets1 expression induces hyperproliferation of the skin epidermis. Immunostaining for epidermal hyperplasia with antibodies to specific markers found in hyperplastic tissues. (A) Staining with anti-keratin 6 (K6) (counterstained with DAPI to detect nuclei). (B) Staining with anti-ΔNp63 (counterstained with nuclear fast red (NFR)). (C) Staining with anti-Ki67 (counterstained with hematoxylin). (D) Staining with anti-PCNA (counterstained with anti-K14). Note that the proliferating cells are located predominantly in the basal layer in both BT and control samples.

As described above, the epidermis of BT mice expressing Ets1 exhibits a dramatic hyperproliferation accompanied by increased numbers of mitotic figures. Several characteristic marker proteins are associated with hyper-proliferative epidermis, but are not normally expressed in the interfollicular epidermis. For instance, the expression of keratin 6 (K6) is normally limited to the hair follicles of mice, but is expressed at high levels in interfollicular epidermis in a variety of hyperplastic and dysplastic conditions. Consistent with this, there was dramatic upregulation of K6 in the interfollicular epidermis of BT mice (Figure 5A). Similar upregulation of K17 was also observed, another keratin that is characteristically expressed by hyperplastic and dysplastic epidermis (data not shown). The enhanced expression of K6 and K17 in BT epidermis suggested the possibility that there might be altered expression of proteins involved in regulating the proliferation of epidermal stem or transit-amplifying cells. One such protein that has been shown to be critical for epidermal stem cell maintenance is the transcription factor p63 [38]. Immunostaining demonstrated that there was a striking increase in the cell population that labeled with antibody against ΔN isoform of p63 (the predominant isoform expressed in skin epidermis) in BT epidermis as compared to control (Figure 5B). Some of these p63+ cells were found in suprabasal layers of BT epidermis, whereas they are only found in the basal layer of wild-type controls. Increased numbers of p63+ cells have also been found in human squamous cell cancers [39], [40] and high levels of p63 expression are correlated with a poor patient prognosis [41], [42].

To more directly test the effects of Ets1 on the proliferation of epidermal keratinocytes, we stained them with antibodies specific for PCNA and Ki67 (Figure 5C–D), proteins expressed only in cells that have entered the cell cycle. The BT epidermis contained many more PCNA+ and Ki67+ cells than did control epidermis, confirming enhanced epidermal proliferation in this tissue. Although the Ets1 transgene was expressed in the more differentiated layers of the epidermis (upper spinous and granular layers), the PCNA and Ki67 labeling was mainly associated with less differentiated layers (basal and lower spinous layers). This observation suggests that expression of Ets1 may not directly induce cell proliferation, but rather induce changes in the differentiation program of the suprabasal cells that cause the basal cells to undergo a dramatic proliferative response. It is possible that the increased proliferation may reflect a secondary response to the compromised barrier function of the epidermis. In addition, some suprabasal keratinocytes also labeled with antibodies to PCNA and Ki67 indicating a supra-basal proliferation.

**Figure 6:**
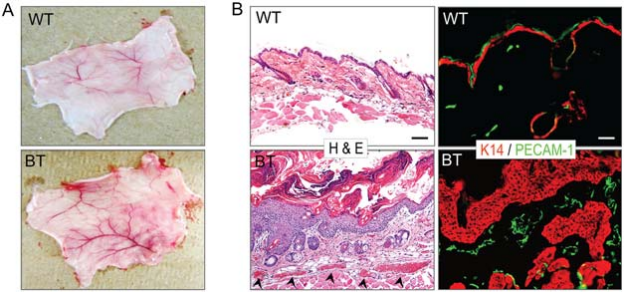
Ets1 expression induces angiogenesis. (A) Macroscopic appearance of the dermal surface of the dorsal skin of wild type and BT mice. (B) Hematoxylin and eosin stained sections of dorsal skin from wild type and BT mice. Note the presence of increased numbers of blood vessels in the dermal compartment of the BT sample (black arrowheads). (C) PECAM-1 (green) staining of skin samples from wild type and BT skin. Sections were stained with antibodies to keratin 14 (K14, red) to reveal epidermal keratinocytes.

While collecting samples of epidermis from BT mice for processing, we noted increased vascularization of the affected skin as evidenced by the presence of numerous, large blood vessels in the dermal tissue (Figure 6A). These vessels were also apparent in hematoxylin and eosin stained sections of the skin epidermis (Figure 6B), which was confirmed by staining for an endothelial marker, PECAM1 (CD31) (Figure 6C). Because the Ets1 transgene is not expressed in endothelial cells (although the endogenous Ets1 gene is expressed in this cell type), this increased angiogenesis is likely due to the release of pro-angiogenic molecules from keratinocytes in the BT epidermis.

### Ets1 affects epithelial homeostasis in a reversible and age-dependent fashion

**Figure 7:**
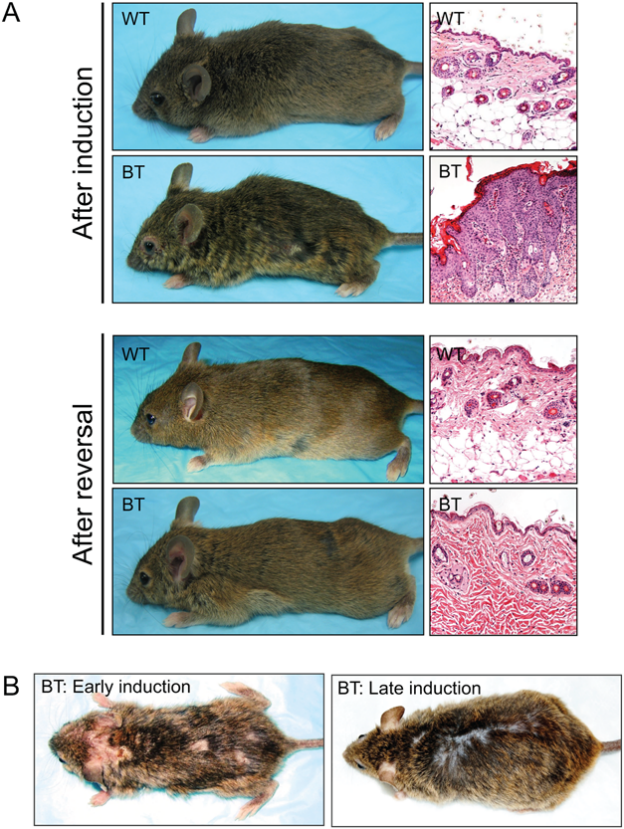
The Ets1 induced phenotype is reversible and age-dependent. (A) Phenotype of BT and control mice at the end of a 3–6 week induction period and again after 6 weeks of transgene suppression on a Dox diet. Note that BT mice appear completely normal after six weeks of rescue. (B) Skin samples taken from BT and control mice at the height of the Ets1 induced phenotype and again after transgene suppression. (C) Comparison of the phenotype of BT mice that were induced for the first time at a young age (early induction) or a late age (late induction). Note the more severe phenotype evident in the early induction.

When BT animals that were severely affected at an early age (4–12 weeks) were returned to a Dox diet to suppress the transgene expression, they recovered completely within six weeks and became indistinguishable from their wild type or single transgenic littermates (Figure 7A). This overall reversal of skin phenotype was also clearly apparent upon microscopic examination of biopsies taken from BT mice before and after Dox rescue (Figure 7B). Within 6 weeks of turning the expression of HA-Ets1 transgene off, the epidermis regressed back to its wild type appearance, but for a residual sebaceous hyperplasia and some dermal fibrosis.

The studies described above were performed on bi-transgenic mice at an early age (4–12 weeks). To test the effects of Ets1 induction in older animals, we withdrew Dox supplementation from animals that were older than six months of age. The animals that underwent a late induction presented with a milder phenotype including minimal hyperplasia and focal hair loss (Figure 7C). Thus, there is an age-dependent severity of the phenotype observed.

### Re-induction of the Ets1 transgene leads to localized lesions

**Figure 8:**
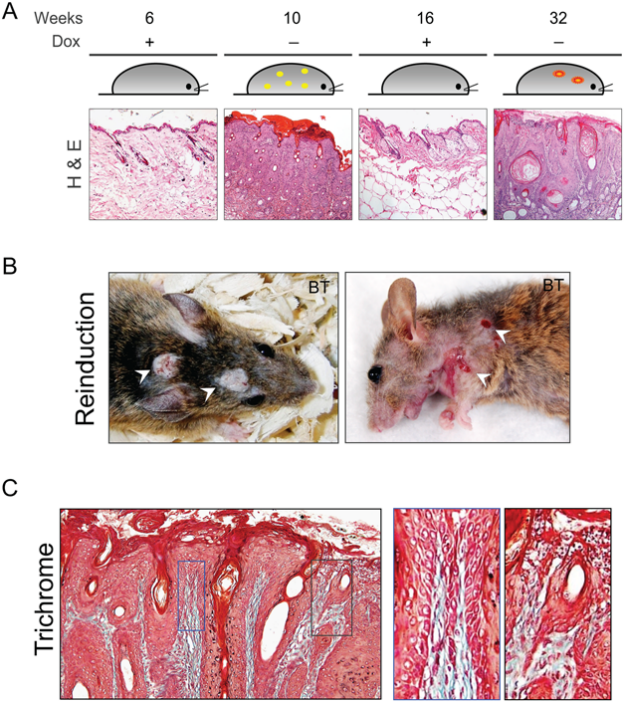
Reinduction of Ets1 in older mice leads to a less severe phenotype. (A) Schematic representation of a typical time course for the re-induction studies. Some BT mice underwent transgene induction at an early age (4–6 weeks) and were then rescued by restoring the Dox diet to suppress transgene expression. The same mice were later reinduced by withdrawing Dox supplementation for an extended time course. After prolonged transgene induction, these re-induced mice developed discrete lesions (papillomatous or ulcerative, white arrows). (B) Hematoxylin and eosin stained sections below the time course indicate typical epidermal morphology at each stage of the induction process. (C) Trichrome staining of the lesions obtained from reinduced BT mice demonstrates downgrowths into the dermis. Boxed areas show regions of thin chords of invading keratinocytes.

To determine whether repeat induction of the transgene would result in a more severe phenotype, we induced young adult BT mice, followed by transgene suppression (by Dox supplementation) and then reinduction at a later age (Figure 8A). The mice chosen for these analyses had a severe visible phenotype in the first induction within a short period, but surprisingly exhibited a more localized phenotype in the second induction only after an extended period of induction (4–7 months). The re-induced mice developed discrete papillomatous or deep ulcerative lesions often with a distinct crateriform appearance, whereas the bulk of the epithelium remained unaffected (Figure 8B). Microscopic examination of biopsies obtained from these lesions revealed the presence of hyperproliferation, dysplasia and deep downgrowths similar to that seen in the primary induction (Figure 8C). Staining for the basement membrane component laminin demonstrated that in some samples there were regions of basement membrane discontinuity (Figure S2). Examination of the differentiation profile of re-induced lesions indicated that they shared properties seen in the epidermis of mice that had undergone an early induction, i.e., they exhibited greatly enhanced proliferation and a partial block to terminal differentiation (Figure S3). Overall, the lesions arising in mice that had undergone secondary induction were less extensive, but potentially more invasive as demonstrated by basement membrane discontinuities.

### Ets1 expression upregulates a panel of genes involved in tumor growth and invasion, including MMP13

To understand the molecular mechanisms that drive the dysplastic changes in the Ets1 BT animals, we analyzed the expression levels of potential Ets1 target genes. These target genes were chosen based on prior studies demonstrating that they were regulated by Ets1 or because they play a critical role in cellular transformation, epithelial-mesenchymal transition (EMT), cell cycle regulation, apoptosis or angiogenesis. For this purpose, we isolated RNA from skin samples of affected BT animals as well as control animals and performed real-time RT-PCR. These analyses allowed us to identify a number of genes whose expression was upregulated in BT epidermis as compared to wild-type or single-transgenic epidermis. The results of these analyses are summarized in Table 1. We detected increased expression of several of the target genes examined including those that are involved in cellular transformation such as Myc and AP-1 proteins (including Junb, Jun (c-Jun) and Fos), cell cycle regulators (including Cdkn1a (p21) and Cdkn2a (p16)) and the anti-apoptotic protein Bcl2l1 (Bcl-xL). In addition to these genes, we also examined a panel of genes that are involved in invasive processes, since the downgrowth of keratinocyte fronts into the dermis was a common feature in all the affected stratified epithelia. Several matrix metallo-proteases (MMPs) including collagenases (MMPs 8 and 13), gelatinase (MMP 9), stromelysins (MMPs 11 and 12), elastase (MMP12) and membrane associated MMPs (MMP 14 and 16) as well as their natural inhibitor Timp-1 were upregulated in BT samples (Table 1). Of the upregulated target genes, MMP13 was the most dramatically induced gene in Ets1 BT mice.

**Table 1:** Gene expression profiles in WT versus BT mice.

Gene Name	Common Name	Fold change in expression
Flt1	VEGF-R1	2.4
Kdr	VEGF-R2	1.67
Tie1	Tie1	0.71
Tek	Tie2	0.57
Angpt2	Angiopoietin-2	1.67
Nos3	eNOS	2
Itgav	αv-Integrin	1.36
Cdh1	E-Cadherin	0.85
Cdh5	VE-Cadherin	0.81
Cdkn1a	p21	4.5
Cdkn2a	p16	6.78
Cdc2l6	CDK11	0.87
Trp53	p53	1.2
Mdm2	Mdm2	1.23
Jun	c-jun	22.0
Junb	JunB	7.26
Fos	c-fos	9.0
Myc	C-myc	2.24
Ddit3	GADD153	1.02
Lmo2	Lmo2	0.83
Vim	Vimentin	0.74
Bcl2l1	Bcl-XL	2.25
Bcl2	Bcl-2	0.6
Casp1	Caspase-1	1.52
Fasl	Fas Ligand	0.08
Tnf	TNF-α	5.5
Pthlh	PTHrP	0.59
Met	c-met	0.95
Plau	uPA	0.64
Serpinb5	Maspin	1.39
Mmp2	Gelatinase A	4.2
Mmp3	Stromelysin-1	3.8
Mmp8	Collagenase-2	151.2
Mmp9	Gelatinase B	43.1
Mmp10	Stromelysin-2	7.4
Mmp11	Stromelysin-3	41.6
Mmp12	Macrophage metalloelastase	13.8
Mmp13	Collagenase-3	770
Mmp14	Membrane type 1-MMP	2.3
Mmp15	Membrane type 2-MMP	0.9
Mmp16	Membrane type 3-MMP	8.0
Mmp17	Membrane type 4-MMP	0.5
Mmp19	Mmp19	0.9
Timp1	Timp1	5.3

MMP13 (also referred to as collagenase-3) is a protease that can cleave native, fibrillar collagens including Type IV collagen, a component of epithelial basement membranes [43]. Expression of MMP13 is very restricted in normal tissues and is not found in skin keratinocytes, except under conditions of wound healing, dysplasia or neoplasia [44], [45], [46], [47], [48]. Moreover, MMP13 expression is considered to be a poor prognostic indicator in SCC [49], [50]. Based on these observations as well as the fact that MMP13 was the most highly-upregulated gene among those we tested, we focused our further analyses on this putative Ets1 target.

**Figure 9:**
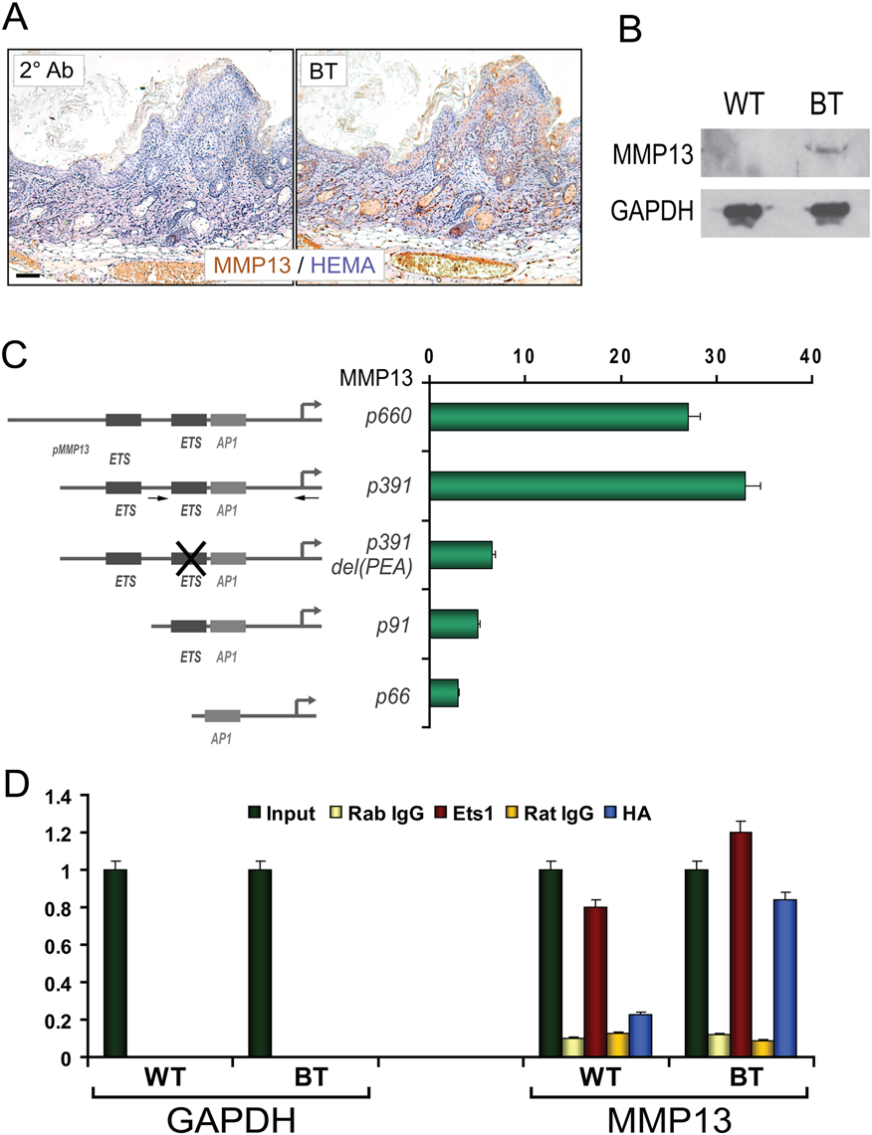
Ets1 expression induces upregulation of MMP13. (A) Immuno-histochemical staining for MMP13 (brown) in the skin of wild-type and BT mice. Sections were counterstained with hematoxylin. Note expression of MMP13 in the invading front (hair follicles and sebaceous glands) and in hyperplastic keratinocytes in the BT sample. (B) Western blot for MMP13 expression in skin extracts of wild-type and BT mice. GAPDH is a loading control. (C) Luciferase assays using different lengths of the mouse MMP13 promoter (MMP13) or an empty vector lacking a promoter (pGL3B) transfected into mouse MK keratinocytes. The MMP13 promoter was transfected with an expression vector driving HA-tagged mouse Ets1 (pCMV-HA-Ets1) or with a control empty vector (pCMV-HA). Results represent the fold induction of luciferase activity in cells transfected with the Ets1 expression vector as compared to empty vector and have been corrected for the activity of the internal control plasmid (pCMVβ-gal). Arrows in the p391 construct indicate the positions of primers used in part D for chromatin immunoprecipitation. (D) Chromatin immunoprecipitation assays to detect association of Ets1 with the endogenous MMP13 promoter in wild-type and BT mice. Antibodies used for immunoprecipitation include anti-Ets1 or a control rabbit IgG and anti-HA or a control rat IgG. Immunoprecipitated chromatin was amplified with primers specific for the mouse MMP13 promoter or for the GAPDH gene. Results represent relative concentrations of products amplified by qPCR.

We performed immunostaining for MMP13 in BT and control mouse skin and found that MMP13 was highly expressed in the BT skin, while there was no staining in the control samples (Figure 9A). This is in agreement with the real time PCR data and also confirms that upregulation of MMP13 is seen in the keratinocyte compartment (although some staining of the dermal compartment was also detected). Expression of MMP13 was particularly evident in the invasive front formed by the proximal portions of hair follicles and sebaceous glands and in the cytoplasmic compartments of hyperplastic keratinocytes. Upregulation of MMP13 expression was confirmed by Western blotting (Figure 9B).

The MMP13 promoter contains three PEA3 motifs (PEA3 sequences are potential Ets binding motifs) and Ets1 has already been shown to bind to one of these sequences by EMSA analysis [16], [48]. These observations suggest that MMP13 is likely to be a direct target of Ets1. To confirm that Ets1 can transactivate the MMP13 promoter, we co-transfected Ets1 and luciferase reporter gene constructs harboring various lengths of MMP13 promoter into a mouse keratinocyte cell line. The reporter gene constructs chosen for these studies have been previously described [48] and include a 660 bp fragment of the mouse MMP13 promoter fused to luciferase (p660) as well as several MMP13 promoter deletions: a 391 bp fragment (p391), a 91 bp fragment (p91) and a 66 bp fragment (p66). Ets1 dramatically transactivated both the 660 bp and 391 bp MMP13 promoter constructs by approximately 30-fold in these cells (Figure 9C), suggesting that the relevant regulatory elements needed for Ets1 transactivation were contained within the first 391 bp of the promoter. In contrast, Ets1 was significantly less effective in transactivating the 91 and 66 bp MMP13 promoters (2–3 fold stimulation of activity by Ets1). These observations suggest that the important Ets1 responsive elements are localized between −91 and −391 bp of the MMP13 promoter. This was confirmed by testing the activity of a mutant form of the p391 promoter (p391 del (PEA)) that contains a short deletion in one of the PEA3 motifs (the −122 bp Ets binding site) (Figure 9C). The deletion construct exhibited responsiveness to Ets1 that was very similar to that of the p91 and p66 promoters. Thus, the Ets1 binding site at −122 bp of the murine MMP13 promoter is essential for maximal transactivation capacity of Ets1.

To confirm that Ets1 can associate with the MMP13 promoter *in vivo*, we performed chromatin immuno-precipitation (ChIP) experiments on dorsal mouse skin epidermis derived from BT and wild type mice using antibodies specific for Ets1 and for the HA-tag on the Ets1 transgene. In wild type mice, the anti-Ets1 antibody precipitated the MMP13 promoter, whereas the HA antibody did not (Figure 9D). In the BT sample, both anti-Ets1 and anti-HA antibodies pulled down the MMP13 promoter. These observations suggest that Ets1 is associated with the MMP13 promoter region in both wild type and BT mice, although the MMP13 gene is only expressed in the BT animals.

## Discussion

The Ets1 transcription factor regulates many important cellular processes including cellular growth and differentiation [51]. High level expression of the Ets1 oncogene has been linked previously to tumor progression, invasion and metastasis in squamous cell tumors as well as various other tumor types [51]. This association with invasion and metastasis is likely to reflect the ability of Ets1 to control the expression of MMP enzymes that function to degrade extracellular matrix components. However, Ets1 is also thought to regulate genes involved in cellular proliferation and survival, suggesting that it might also play a role in the earlier stages of carcinogenesis. To determine the precise biological role of Ets1 in tumor formation and progression, we developed a transgenic mouse model that allows us to induce the expression of an Ets1 transgene in a spatially and temporally-restricted manner.

### Ets1 drives dysplastic changes in stratified squamous epithelium

Using an inducible transgenic model system, we have demonstrated that expression of the Ets1 oncogene in the differentiating layers of mouse stratified squamous epithelium leads to a dramatic epithelial phenotype, visibly apparent by the appearance of crusting, flaky skin and open sores. Dysregulated Ets1 expression induced a wide spectrum of progressive premalignant changes such as those induced by chemical carcinogens, activated oncogenes, or loss of tumor suppressors in well-established mouse models of squamous cell carcinogenesis. The dysplastic phenotype includes increased keratinocyte proliferation, decreased keratinocyte terminal differentiation and the presence of mitoses (some of which were abnormal) in the suprabasal layers of the epidermis. The dermal responses include increased vascularization and leukocytic infiltration.

The inhibition of expression of terminal differentiation markers such as involucrin and loricin caused by Ets1 induction likely leads to a severe defect in epidermal barrier formation. Indeed, the phenotype induced by Ets1 transgene expression is similar to that seen in the adult skin of other strains of mice reported to develop barrier defects (e.g., transglutaminase-1 deficient skin or matriptase over-expressing skin) [52], [53]. Like these strains, Ets1 BT mice develop extensive skin hyperplasia, abnormal terminal differentiation and hyperkeratosis. However, Ets1 BT mice also develop deep down-growths of the epidermis into the dermal compartment, which have not been reported in these other strains. These down-growths may be related to the high expression of several MMP family members by Ets1 BT mice. The defect in epidermal barrier function in the BT mice could trigger a hyper-proliferative state as an attempt to restore normal barrier function. This would be consistent with the observation that the majority of Ki67+ and PCNA+ cells are found in the basal layer rather than the suprabasal layers, where Ets1 is expressed. In contrast, dysplastic changes including abnormal terminal differentiation and the presence of abnormal mitoses were associated with the supra-basal layers where the Ets1 transgene is expressed, suggesting a direct effect of Ets1 on these parameters. Together these results indicate that Ets1 may have an early role in cancer progression in addition to its more established role in tumor metastasis. Despite the extensive alterations in cutaneous architecture, the molecular and the consequent structural changes in the inter-follicular epidermis are completely reversible when Ets1 expression is suppressed by re-instating Dox treatment. This reversal of the dysplastic phenotype is reminiscent of human SCC precursor lesions such as actinic keratosis and Bowen's disease, which can undergo spontaneous regression.

Importantly, these changes in the epidermis occur in the absence of any tumor promoting chemicals or UV radiation. Moreover, the fact that the entire skin epidermis is affected (in young animals), that the phenotype develops very rapidly after induction (within 3–6 weeks) and that the phenotype is completely reversible suggest that additional genetic hits to other oncogenes or tumor suppressor genes are not required. Hence, over-expression of Ets1 alone is sufficient to cause severe epidermal dysplasia.

The dysplastic phenotype induced by Ets1 expression was stronger in animals induced at a young age (less than 3 months of age). The reason for this age-dependent affect is not yet clear, however, it may potentially be due to lower levels of expression of the transgene in older animals or to changes in the physiology of the epidermis between young and old mice. When we induced BT mice at a young age followed by suppression of Ets1 expression and re-induction at an older age, we noted that the phenotype in the second induction was also overall weaker. This weaker phenotype allowed a longer induction period after which discrete ulcerative or papillomatous lesions arose in these re-induced mice. The appearance of discrete lesions in an otherwise weakly affected epithelium and the need for a long induction period strongly suggests that additional genetic hits must occur in these tissues to promote lesion formation. Alternatively, it is possible that stochastic down-regulation of the Ets1 transgene expression via epigenetic changes could result in the expression of the transgene in discrete patches in the adult.

### Cells already committed to differentiation are targets of Ets1 action

Oncogenic mutations in stem cells have been implicated in the development of skin tumors [54], [55]. Epidermal stem cells are thought to reside in the hair follicle bulge region as well as being scattered throughout the basal layer of the interfollicular epidermis. Interestingly, in our transgenic model system, Ets1 overexpression is not targeted to the epidermal stem cells, but rather to keratinocytes that have already committed to differentiation. This is in contrast to most other transgenic models of SCC, where transgene expression is targeted to the basal proliferative layer (e.g., oncogenic Ras, [56]). Indeed, when the Ets1 transgene is expressed in the basal compartment of the epidermis (including epidermal stem cells) using a keratin 5 (K5) tTA transgenic line, no phenotype is evident in adult BT mice (our unpublished data). Interestingly expression of c-myc in the differentiated layers of the skin also results in hyperproliferation and formation of papillomas [57], whereas targeting c-myc to the basal proliferative layer does not promote keratinocyte proliferation or papilloma formation [58]. Thus, Ets1 and c-myc appear to share a property that allows them to induce pre-neoplastic changes in epidermal cells that have already committed to terminal differentiation.

### Ets1 regulates the expression of a variety of genes implicated in tumorigenesis

Mice expressing the Ets1 transgene exhibit upregulation of many known and potential Ets1 target genes involved in proliferation, survival and apoptosis. These genes include proteases, cell cycle regulators, anti-apoptotic proteins and transcription factors. The phenotype arising in the induced BT mice likely reflects the combined action of several of these genes. For instance, upregulation of Myc in the suprabasal epidermal layers has been shown to lead to a similar epidermal phenotype characterized by reversible epidermal papillomatosis and sebaceous gland enlargement [57], [59]. Thus, upregulation of Myc could contribute to multiple aspects of the dysplastic phenotype observed in Ets1 BT mice. Another group of proteins that could potentially contribute to Ets1 induced dysplastic epidermal changes is the AP-1 family. AP-1 proteins control transcriptional activation or suppression of a number of genes involved in proliferation, differentiation and transformation. Of the AP-1 proteins that are upregulated in Ets1 BT skin, Jun and Fos have been shown to contribute to squamous carcinogenesis [60], [61]. Moreover, AP-1 transcription factors along with Ets proteins can exert a combinatorial control of expression of several genes that include importantly metalloproteinases.

Matrix metalloproteinases appear to be chief effectors of Ets1 induced epithelial alterations [51]. Several MMPs have been shown to be direct targets of Ets proteins and are often co-regulated by AP-1 proteins. Various classes of metalloproteinases such as collagenases (MMPs 8 and 13), gelatinases (MMPs 2 and 9), stromelysins (MMPs 3, 10 and 11), metalloelastase (MMP12) and membrane-type MMPs (MMPs 14 and 16) are upregulated in Ets1 BT skin. This suggests that several biological processes that accompany Ets1 induced dysplasia could be attributed to the function of MMPs. We focused our attention on the most highly upregulated target gene, matrix metalloprotease 13 (MMP13). We demonstrate that Ets1 directly regulates expression of the MMP13 gene and can be detected at the promoter region. Interestingly, Ets1 was associated with the promoter region of MMP13 in both BT and control skin samples. This may suggest that Ets1 is constitutively associated with the promoter of this gene, but is inactive unless it becomes phosphorylated or that it requires the recruitment of an inducible co-factor to mediate full induction of the gene. -

MMP enzymes including MMP13 are best known for their ability to degrade a wide variety of extracellular matrix substrates including collagen, fibronectin, laminin, perlecan, tenascin C and fibrillin [43], [62]. By degrading extracellular matrix components, MMPs can promote the invasion of cells into the surrounding tissues. Moreover, degradation of extracellular matrix may lead to the release of latent growth factors and angiogenic factors, promoting tumor cell proliferation. Furthermore, numerous studies have detected degradation of key epithelial adhesion proteins, such as E-cadherin and desmogleins, by members of the MMP family [63], [64], [65], [66], [67], [68], suggesting that MMP activity can result in diminished keratinocyte adhesion to neighboring cells. Together these activities of MMP proteins can promote keratinocyte proliferation, migration and invasion.

-MMP proteins have the ability to promote dysplastic changes in tissues, even when they are expressed alone in the absence of other genetic changes or tumor inducing chemicals or UV radiation[63], [69], [70], [71], [72]. In addition, over-expression of matriptase, a protease that has been suggested to be an upstream activator of MMP3 [73], induces squamous cell carcinoma in the skin [52]. Thus, expression or activation of numerous MMP proteins can promote hyperplastic and neoplastic changes in epithelial tissues, supporting the idea that overexpression of MMPs may be important to the development of pre-neoplastic and neoplastic alterations in Ets1 BT mice. It is likely that MMPs cooperate with other genes induced by Ets1 overexpression (such as myc and AP-1 family members) to promote the dysplastic changes.

### The role of Ets1 in keratinocyte differentiation and future prospects

In addition, to promoting proliferation and invasion of skin keratinocytes, Ets1 expression also blocked their terminal differentiation program. The block to terminal differentiation likely results in impaired barrier formation in the skin and is likely the cause of perinatal mortality for Ets1 BT mice that undergo transgene induction during embryonic development. The effect of Ets1 in blocking terminal differentiation may arise from its ability to interfere with the transcriptional programs that drive the differentiation process. For instance, we have recently shown that Ets1 physically interacts with the transcription factor Blimp-1 to inhibit its function [74]. Blimp-1 is important for the final differentiation step of keratinocytes from the granular layer to the cornified layer [75]. By interfering with Blimp-1 or other transcription factors, Ets1 may prevent their function and thereby block the terminal differentiation of the epidermis.

In summary, using an inducible transgenic mouse model, we have demonstrated that expression of the Ets1 transcription factor leads to a dramatic hyperplastic and dysplastic phenotype in skin epithelium of young mice in the apparent absence of other genetic lesions. This represents the first report that Ets1 can play a major role in the early stages of the carcinogenic process. Together, these studies extend our understanding of the role of Ets1 in squamous cell tumors and suggest mechanisms by which Ets1 can promote cellular transformation and tumor progression. Our data described here strongly suggest that in a variety of human carcinomas, over-expression of Ets1 may not just be a prognostic indicator, but play an important causal role that can be exploited for targeted therapy. In the future, our transgenic model system can be used to induce Ets1 in any tissue or cell type by utilizing the appropriate tetracycline-transactivator lines, and hence will prove useful in understanding the role of Ets1 in regulating development, differentiation and oncogenic conversion in a variety of tissues.

## Materials and Methods

### Ethics Statement

All mouse experiments were approved by the State University of New York at Buffalo IACUC committee and carried out in accordance with relevant national guidelines.

### Generation and analysis of transgenic mice

The Ets1 doxycycline inducible construct was generated by cloning full length mouse Ets1 cDNA with a 5′ HA tag into the pTRE-Tight plasmid (BD Biosciences Clontech). The inducible Ets1 construct was micro-injected into fertilized mouse oocytes derived from a mixed genetic background (C3Hf/HeRos×C57BL/10 Rospd). Six transgenic lines were generated in these initial injections. The following primers were used to genotype the transgenic mice: forward (5′-ATAGTTGTGACCGCCTCACC -3′) and reverse (5′- GGGAGGTGTGGGAGGTTTT-3′). The founders were then crossed to INV-tTA mice (a kind gift from Dr. Julia Segre, National Human Genome Research Institute, Bethesda) to determine expression of the transgene. Inv-tTA mice were on an FVB/N genetic background and BT mice derived from these crosses were subsequently backcrossed to FVB/N mice for several generations.

### Antibodies Used

The following primary antibodies were used for Western blotting, immunofluorescence and/or immunohistochemistry: rat monoclonal anti-HA (clone 3F10, Roche Applied Sciences), rabbit polyclonal anti-Ets1 (N-276, Santa Cruz Biotechnology), mouse monoclonal anti-GAPDH (clone 6C5, Chemicon International), goat polyclonal anti-MMP13 (W-16, Santa Cruz Biotechnology), mouse monoclonal anti-Ki67 (NCL-Ki67p, Novocastra), rat monoclonal anti-β4 integrin (CD104, clone 346-11A, BD Biosciences), rat monoclonal anti-PECAM1 (CD31, clone MEC-13.3, BD Biosciences), rabbit polyclonal anti-laminin I (CL54851AP, Cedarlane Laboratories) and mouse monoclonal anti-PCNA (clone PC10, Dakocytomation). A rabbit polyclonal anti-ΔNp63 antibody (RR-14, developed in the laboratory of Dr. Satrajit Sinha) was also used. In addition, several polyclonal rabbit primary antibodies specific for keratinocyte marker proteins (keratin 1, keratin 5, keratin 6, keratin 10, keratin 14, involucrin and loricrin) were generous gifts of Dr. Julia Segre (National Human Genome Research Institute, National Institutes of Health). The rabbit polyclonal antibody against keratin 17 (K17) was a gift of Dr. Pierre Coloumbe (Johns Hopkins University School of Medicine).

### Western blotting

Whole tissue lysates were prepared using Laemmli sample buffer (BioRad Laboratories) or RIPA buffer from BT and control mouse dorsal skins. Equal amounts of lysates were loaded on 10% SDS-polyacrylamide gels and transferred to Immun-Blot PVDF membrane (BioRad Laboratories). After blocking, the membranes were incubated in the specific primary antibodies: anti-HA (1∶2000), anti-Ets1 (1∶1000), anti-GAPDH (1∶10000) or anti-MMP13 (1∶200). Antibodies against keratinocyte markers – keratin10, keratin14, involucrin and loricrin -were used at a dilution of 1∶5000. After washing, the membranes were incubated with specific horseradish peroxidase (HRP) conjugated secondary antibodies at 1∶20,000 dilution. Specific bands were detected by chemiluminescence (KPL or Pierce).

### Immunohistochemistry and Immunofluorescence

For histological analyses, skin samples from various regions of the body were fixed in 4% paraformaldehyde and embedded in paraffin. 4-µm thick sections were then stained with hematoxylin and eosin or Masson's trichrome reagent. For immunohistochemistry, 4-µm thick skin sections were deparaffinized and subjected to antigen retrieval by heating. The sections were then immuno-stained with specific primary antibodies - anti-HA (1∶300), anti-ΔNp63 (1∶300), anti-Ki67 (1∶200), anti-Ets1 (1∶150), anti-MMP13 (1∶40), anti-keratin 1 (1∶500), anti-keratin 5 (1∶500), anti-keratin 6 (1∶500), anti-involucrin (1∶500), and anti-loricrin (1∶500) - using the Vectastain ABC kit (Vector labs). Diaminobenzidine (DAB) or Vector Blue (Vector Labs) was used as the enzyme substrate and counter-stained with hematoxylin or Nuclear Fast Red (NFR).

For immunofluorescence, 5-µm thick OCT embedded fresh frozen sections were fixed in ice-cold methanol. After blocking, the sections were incubated with anti-keratin6, anti-keratin10, anti-keratin14, anti-loricrin, anti-β4 Integrin or anti-PECAM1 antibodies at 1∶500 dilution or with anti-Laminin or anti-PCNA antibodies at 1∶100 dilution. The sections were then washed and incubated with Alexa-Fluor 568 coupled anti-rabbit IgG (1∶750), Alexa-Fluor 488 coupled anti-rat IgG (1∶500) or FITC coupled anti-mouse IgG (1∶500) antibodies and counterstained with DAPI (4′,6-diamidino-2-phenylindole). The immunostained sections were mounted in 80% glycerol in PBS, viewed and photographed at room temperature with an Axiophot Zeiss microscope, equipped with a Hamamatsu ORCA-ER camera linked to SPOT software for image capture. The contrast and brightness of the images were adjusted using the Adobe Photoshop and ImageJ software.

### Quantitative PCR Analysis

Total RNAs were isolated using Trizol (Invitrogen) and treated with DNAse I Turbo (Ambion) to remove genomic DNA contamination. Equivalent quantities of RNA were reverse transcribed using iScript kit (BioRad) and real-time PCR was performed in the ICycler system (BioRad), using iQ SYBR green supermix (BioRad) and gene specific primers. Differences between BT samples and controls were normalized to expression of the ubiquitously-expressed gene β_2_-microglobulin and determined using the 2^−ΔCt^ method.

### Reporter gene assays

For transient transfections, mouse MK1 keratinocyte cells were plated at a density of 1.2×10^5^ cells/well in 6 well plates and were transfected using Optifect (Invitrogen) or Fugene 6 reagent (Roche Diagnostics) at a density of 40% according to the manufacturer's instructions. The cells were transfected with 0.5 µg/well of each of the following plasmids: pGL3-basic or various lengths of MMP13 promoter cloned into the pGL3-basic plasmid: −660 bp, −391 bp, −391 bp ΔPEA, −91 bp, and −66 bp relative to transcription start site (generous gifts from Dr. Jeffrey M. Davidson, Vanderbilt University, Nashville and Dr. Keith Kirkwood, University of Michigan, Ann Arbor). Cells were also co-transfected with the empty pCMV-HA vector (Clontech) or pCMV-HA-Ets1 (containing an HA-tagged version of mouse Ets1) and with pCMV-LacZ (0.25 µg/well) as an internal control. Cells were harvested 48 hours post transfection in Reporter Lysis buffer (Promega) and luciferase assays were performed. β-galactosidase values were measured using the Galacton Plus kit (Applied Biosystems). The luciferase values were normalized to β-galactosidase levels to correct for transfection efficiency. Reporter assays were performed in duplicates of at least three independent experiments and expressed as the means±S.D.

### Chromatin Immunoprecipitation

Dorsal skins from wild-type and BT adult mice were de-epilated and treated with ammonium thiocyanate solution to separate the epidermis from the underlying dermis. The epidermal cells were disaggregated and fixed in 1% formaldehyde for 15 mins at RT. After washing, the cells were sonicated to fragment chromatin. The chromatin was then immunoprecipitated using the Chromatin Immunoprecipitation Assay kit (Upstate Biotechnology) and the following antibodies: anti-HA, anti-Ets1, isotype-control rat IgG or isotype control rabbit IgG. Immunoprecipitated samples were analyzed by PCR using primers designed to the minimal promoter of mouse MMP13: forward 5′ TCCATTTCCCTCAGATTCTGCCAC-3′ and reverse 5′-CAGCAGTGCCTGGAGTCTCT-3′. As a control, PCR was also performed using primers that recognize the minimal promoter of mouse GAPDH.

### Online Supplemental Material

Hematoxylin and eosin staining and immuno-staining results presented in the online supplementary figures were collected using the same techniques and reagents as described above.

## Supporting Information

Figure S1 Ets1 is not essential for epidermal development. (A) Ets1 knockout mouse demonstrating areas of white spotting (non-pigmented hair and skin) (B) Hematoxylin and eosin stained section of adult dorsal skin from an Ets1 knockout mouse, showing normal histology. C. High power view of boxed area in B.
(1.48 MB TIF)

Figure S2 Loss of basement membrane integrity in re-induced lesions. Immunostaining for laminin I (red) shows breaks in basement membrane (arrows) in cutaneous lesions of the re-induced BT mice. Tissues are counterstained with K14 (to mark keratinocytes) and DAPI (to mark nuclei).
(1.87 MB TIF)

Figure S3 Enhanced keratinocyte proliferation and block to terminal differentiation in the re-induced tumors. Detection of stage-specific keratinocyte differentiation and proliferation markers in re-induced lesions by immunostaining (DAB staining, brown). The lesions display enhanced expression of early (keratin 5, K5) and intermediate differentiation (keratin 1, K1 and involucrin, inv) markers and decreased expression of the late differentiation marker loricrin (Lor). There is also enhanced expression of proliferation markers including- Ets1, keratin 6 (K6), Ki67, and DeltaNp63.
(1.96 MB TIF)
