# Supplementary figures and images for: Ets1 Induces Dysplastic Changes When Expressed in Terminally-Differentiating Squamous Epidermal Cells
Source: PLoS One. 2009 Jan 14;4(1):e4179. doi: 10.1371/journal.pone.0004179 (PMC2615206; doi:10.1371/journal.pone.0004179)

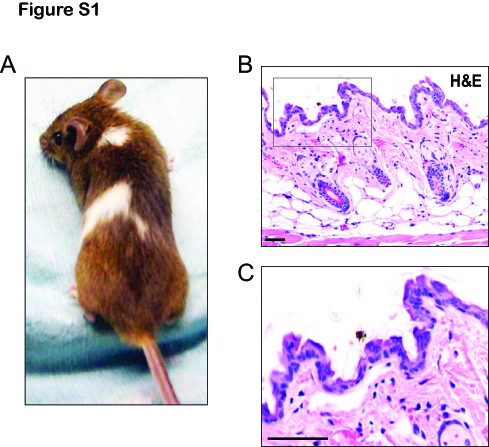

Supplement: Figure S1 — Ets1 is not essential for epidermal development. (A) Ets1 knockout mouse demonstrating areas of white spotting (non-pigmented hair and skin) (B) Hematoxylin and eosin stained section of adult dorsal skin from an Ets1 knockout mouse, showing normal histology. C. High power view of boxed area in B. (1.48 MB TIF) [file pone.0004179.s001.tif]

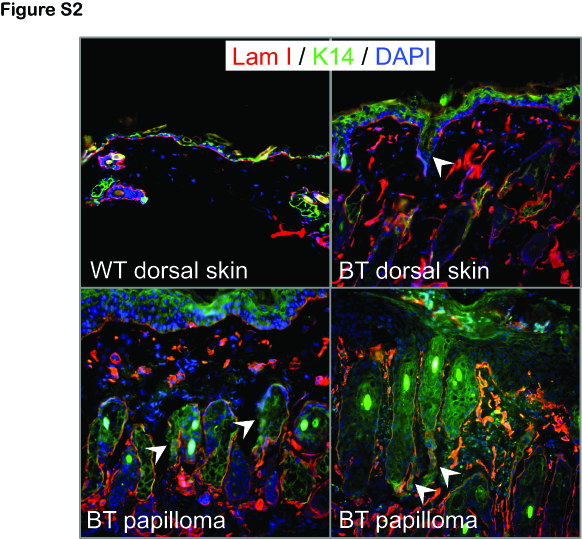

Supplement: Figure S2 — Loss of basement membrane integrity in re-induced lesions. Immunostaining for laminin I (red) shows breaks in basement membrane (arrows) in cutaneous lesions of the re-induced BT mice. Tissues are counterstained with K14 (to mark keratinocytes) and DAPI (to mark nuclei). (1.87 MB TIF) [file pone.0004179.s002.tif]

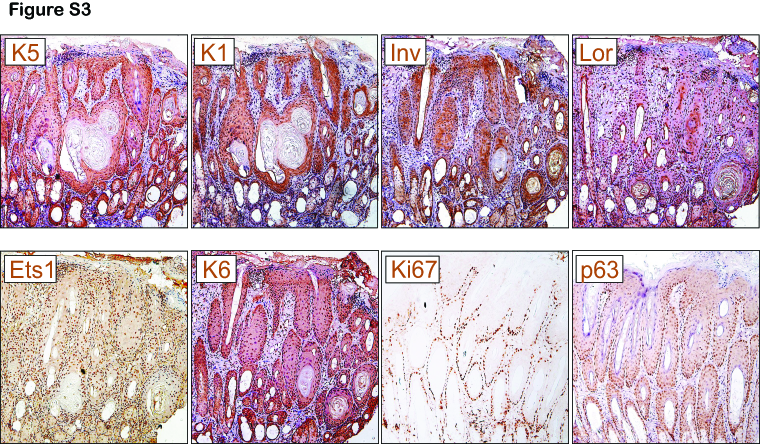

Supplement: Figure S3 — Enhanced keratinocyte proliferation and block to terminal differentiation in the re-induced tumors. Detection of stage-specific keratinocyte differentiation and proliferation markers in re-induced lesions by immunostaining (DAB staining, brown). The lesions display enhanced expression of early (keratin 5, K5) and intermediate differentiation (keratin 1, K1 and involucrin, inv) markers and decreased expression of the late differentiation marker loricrin (Lor). There is also enhanced expression of proliferation markers including- Ets1, keratin 6 (K6), Ki67, and DeltaNp63. (1.96 MB TIF) [file pone.0004179.s003.tif]
